# *trans*-Diaqua­bis­[5-carb­oxy-2-(3-pyrid­yl)-1*H*-imidazole-4-carboxyl­ato-κ^2^ *N* ^3^,*O* ^4^]cobalt(II)

**DOI:** 10.1107/S1600536811029618

**Published:** 2011-08-02

**Authors:** Qing-Guang Zhan

**Affiliations:** a School of Chemistry and Environment, South China Normal University, Guangzhou 510006, People’s Republic of China, and South China Normal University, Key Laboratory of Technology on Electrochemical Energy Storage and Power Generation in Guangdong Universities, Guangzhou 510006, People’s Republic of China

## Abstract

In the title complex, [Co(C_10_H_6_N_3_O_4_)_2_(H_2_O)_2_], the Co^II^ atom is located on an inversion centre and displays a distorted octa­hedral coordination geometry defined by two *N*,*O*-bidentate ligands in the equatorial plane and two water mol­ecules in the axial positions. The conformation is stabilized by intra­molecular O—H⋯O hydrogen bonds. Inter­molecular N—H⋯O hydrogen bonds link the mol­ecules into chains, which are further connected by inter­molecular O—H⋯O and O—H⋯N hydrogen-bonding inter­actions, forming a two-dimensional supra­molecular network parallel to (110).

## Related literature

For general background to the design and synthesis of coordination polymers based on 1*H*-imidazole-4,5-dicarb­oxy­lic acid, see: Gu *et al.* (2010 ▶); Wang *et al.* (2010 ▶). For related complexes with 5-carb­oxy-2-(3-pyrid­yl)-1*H*-imidazole-4-carboxyl­ate, see: Chen (2008 ▶); Liu *et al.* (2009 ▶); Jing *et al.* (2010 ▶, 2011 ▶); Zhou *et al.* (2011 ▶).
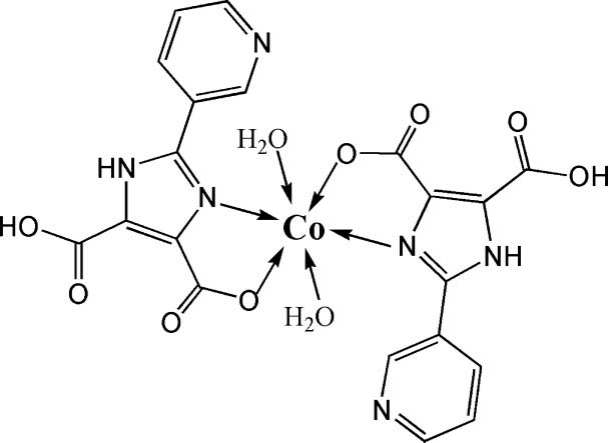


## Experimental

### 

#### Crystal data

[Co(C_10_H_6_N_3_O_4_)_2_(H_2_O)_2_]
*M* *_r_* = 559.32
Triclinic, 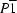

*a* = 7.0240 (9) Å
*b* = 8.8770 (12) Å
*c* = 9.3240 (12) Å
α = 81.598 (2)°
β = 83.290 (2)°
γ = 67.755 (2)°
*V* = 531.12 (12) Å^3^
*Z* = 1
Mo *K*α radiation
μ = 0.88 mm^−1^
*T* = 298 K
0.32 × 0.28 × 0.26 mm

#### Data collection

Bruker SMART APEXII CCD area-detector diffractometer
Absorption correction: multi-scan (*SADABS*; Bruker, 2005 ▶) *T*_min_ = 0.765, *T*_max_ = 0.803
2936 measured reflections
2052 independent reflections
1589 reflections with *I* > 2σ(*I*)
*R*_int_ = 0.017

#### Refinement

*R*[*F* ^2^ > 2σ(*F* ^2^)] = 0.044
*wR*(*F* ^2^) = 0.104
*S* = 1.06
2052 reflections
170 parameters
H-atom parameters constrained
Δρ_max_ = 0.30 e Å^−3^
Δρ_min_ = −0.33 e Å^−3^

### 

Data collection: *APEX2* (Bruker, 2005 ▶); cell refinement: *SAINT* (Bruker, 2005 ▶); data reduction: *SAINT*; program(s) used to solve structure: *SHELXTL* (Sheldrick, 2008 ▶); program(s) used to refine structure: *SHELXTL*; molecular graphics: *SHELXTL*; software used to prepare material for publication: *SHELXTL*.

## Supplementary Material

Crystal structure: contains datablock(s) I, global. DOI: 10.1107/S1600536811029618/rz2627sup1.cif

Structure factors: contains datablock(s) I. DOI: 10.1107/S1600536811029618/rz2627Isup2.hkl

Additional supplementary materials:  crystallographic information; 3D view; checkCIF report

## Figures and Tables

**Table 1:** Hydrogen-bond geometry (Å, °)

*D*—H⋯*A*	*D*—H	H⋯*A*	*D*⋯*A*	*D*—H⋯*A*
O3—H3⋯O2	0.82	1.65	2.465 (3)	176
N2—H2⋯O4^i^	0.86	2.00	2.840 (3)	166
O5—H5*A*⋯O3^ii^	0.85	2.07	2.918 (3)	173
O5—H5*B*⋯N3^iii^	0.85	2.02	2.784 (3)	150

Symmetry codes: (i) 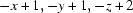; (ii) 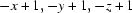; (iii) 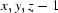.

**Fig. 1.**
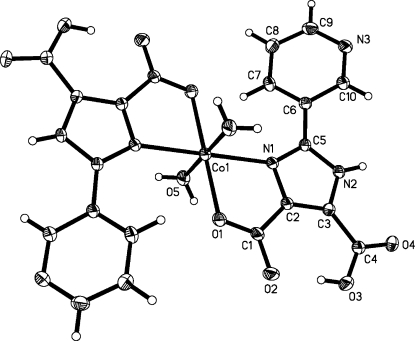
The molecular structure of the title compound, with displacement ellipsoids drawn at the 30% probability level. Unlabelled atoms are related to the labelled atoms by the symmetry operation 2-x, -y, 1-z.

**Fig. 2.**
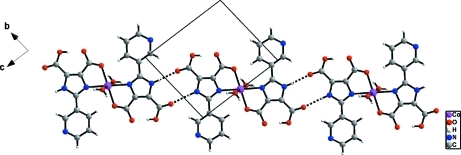
Crystal packing of the title compound viewed down the a axis, showing the one-dimensional chain structure. Hydrogen bonds are shown as dashed lines.

**Fig. 3.**
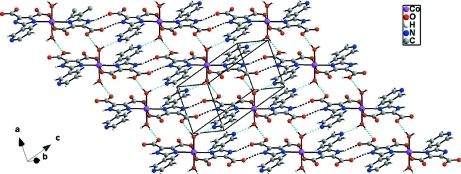
Crystal packing of the title compound showing the two-dimensional hydrogen-bonding network (dashed lines).

## supplementary crystallographic information

### Comment

In recent years, the design and synthesis of novel metal-organic coordination polymers based on *N*-heterocyclic carboxylic acids have provoked much attention owing to their structure diversity and their potential applications as functional materials. Particular attention has been paid to the 1H-imidazole-4,5-dicarboxylic acid (H_3_IDC) ligand, because it can coordinate with metal ions in diverse coordination fashions to produce a series of complexes with different structures and interesting properties (Gu *et al.*, 2010; Wang *et al.*, 2010). In this work, a very close analogue ligand of H_3_IDC, 5-carboxy-2-(3-pyridyl)-1H-imidazole-4-carboxylate (H_3_PyIDC), has been chosen to prepare new coordination polymers. Up to now, only two 0D clusters (Chen, 2008; Liu *et al.*, 2009), one 1D chain (Jing *et al.*, 2011), one 2D layer (Zhou *et al.*, 2011), and one 3D network (Jing *et al.*, 2010) constructed by the H_3_PyIDC ligand have been reported. For example, Chen (2008) and Liu *et al.* (2009) have described the structures of the mononuclear complexes [Mn(H_2_PyIDC)_2_(H_2_O)_2_] and [Fe(H_2_PyIDC)_2_(H_2_O)_2_], in which both the manganese(II) and iron(II) ions show octahedral coordinations with the H_2_PyIDC ligands. A new complex [Co(H_2_PyIDC)_2_(H_2_O)_2_], (I), which is isostructural with the manganese(II) and iron(II) analogues, is presented in this paper.

As illustrated in Fig. 1, the molecule of (I) is a discrete neutral monomer, with an asymmetric unit that contains one-half of the [Co(H_2_PyIDC)_2_(H_2_O)_2_] formula unit. The Co atom lies on an inversion center and is six-coordinated with two nitrogen and two oxygen atoms from two chelating H_2_PIDC ligands in the equatorial plane, and two coordinated water molecules in axial positions, forming a slightly distorted octahedral geometry. The Co–N (2.215 (2) Å) and Co–O nond distances (2.061 (2)–2.098 (2)Å) are similar to the M–O and M–N bond lengths ( M = Mn, Fe ) observed in the corresponding isotypic structures. The conformation of the complex is stabilized by intramolecular O—H···O hydrogen bonds. In the crystal structure, intermolecular N–H···O hydrogen bonds (Table 1) link the molecules into one-dimensional chains as shown in Fig. 2. The chains are further connected by two types of hydrogen bonds O–H···O and O–H···N involving the coordinated water molecule, the carboxylate group and the uncoordinated pyridine N atoms, resulting in a two-dimensional supramolecular network (Fig. 3).

### Experimental

A mixture of CoCl_2_.6H_2_O (47.6 mg, 0.2 mmol), H_3_PyIDC (46.6 mg, 0.2 mmol), 8 ml H_2_O, and 0.1 mL Et_3_N was sealed in a 15 mL Teflon-lined stainless steel autoclave, heated at 443 K for 72 h, and then slowly cooled to room temperature at a rate of 5 K h^-1^. Brown block-shaped crystals of (I) were obtained with a yield of 28% after washing with distilled water and drying in air.

### Refinement

Water H atoms were located in a difference Fourier map and refined with distance restraints of O—H = 0.85 Å and *U*_iso_(H) = 1.2 *U*_eq_(O). Other H atoms were fixed geometrically and treated as riding with C—H = 0.93 Å (aromatic), O—H = 0.82 Å and N—H = 0.86 Å with *U*_iso_(H) = 1.2 *U*_eq_(C, N) or U_iso_(H)= 1.5 U_eq_(O).

### Crystal data

**Table:**

[Co(C_10_H_6_N_3_O_4_)_2_(H_2_O)_2_]	*Z* = 1
*M**_r_* = 559.32	*F*(000) = 285
Triclinic, *P*1	*D*_x_ = 1.749 Mg m^−^^3^
Hall symbol: -P 1	Mo *K*α radiation, λ = 0.71073 Å
*a* = 7.0240 (9) Å	Cell parameters from 860 reflections
*b* = 8.8770 (12) Å	θ = 2.2–24.9°
*c* = 9.3240 (12) Å	µ = 0.88 mm^−^^1^
α = 81.598 (2)°	*T* = 298 K
β = 83.290 (2)°	Block, brown
γ = 67.755 (2)°	0.32 × 0.28 × 0.26 mm
*V* = 531.12 (12) Å^3^	

### Data collection

**Table:**

Bruker SMART APEXII CCD area-detector diffractometer	2052 independent reflections
Radiation source: fine-focus sealed tube	1589 reflections with *I* > 2σ(*I*)
graphite	*R*_int_ = 0.017
phi and ω scans	θ_max_ = 26.0°, θ_min_ = 2.2°
Absorption correction: multi-scan (*SADABS*; Bruker, 2005)	*h* = −8→8
*T*_min_ = 0.765, *T*_max_ = 0.803	*k* = −10→6
2936 measured reflections	*l* = −10→11

### Refinement

**Table:**

Refinement on *F*^2^	Primary atom site location: structure-invariant direct methods
Least-squares matrix: full	Secondary atom site location: difference Fourier map
*R*[*F*^2^ > 2σ(*F*^2^)] = 0.044	Hydrogen site location: inferred from neighbouring sites
*wR*(*F*^2^) = 0.104	H-atom parameters constrained
*S* = 1.06	*w* = 1/[σ^2^(*F*_o_^2^) + (0.0385*P*)^2^ + 0.3865*P*] where *P* = (*F*_o_^2^ + 2*F*_c_^2^)/3
2052 reflections	(Δ/σ)_max_ < 0.001
170 parameters	Δρ_max_ = 0.30 e Å^−^^3^
0 restraints	Δρ_min_ = −0.33 e Å^−^^3^

### Special details

**Table:**

Geometry. All esds (except the esd in the dihedral angle between two l.s. planes) are estimated using the full covariance matrix. The cell esds are taken into account individually in the estimation of esds in distances, angles and torsion angles; correlations between esds in cell parameters are only used when they are defined by crystal symmetry. An approximate (isotropic) treatment of cell esds is used for estimating esds involving l.s. planes.
Refinement. Refinement of F^2^ against ALL reflections. The weighted R-factor wR and goodness of fit S are based on F^2^, conventional R-factors R are based on F, with F set to zero for negative F^2^. The threshold expression of F^2^ > 2sigma(F^2^) is used only for calculating R-factors(gt) etc. and is not relevant to the choice of reflections for refinement. R-factors based on F^2^ are statistically about twice as large as those based on F, and R- factors based on ALL data will be even larger.

### Fractional atomic coordinates and isotropic or equivalent isotropic displacement parameters (Å^2^)

**Table:**

	*x*	*y*	*z*	*U*_iso_*/*U*_eq_	
Co1	1.0000	0.0000	0.5000	0.0352 (2)	
O3	0.5734 (4)	0.6730 (3)	0.6242 (2)	0.0470 (6)	
H3	0.6370	0.6154	0.5601	0.071*	
N2	0.6548 (4)	0.2945 (3)	0.8556 (2)	0.0313 (6)	
H2	0.5996	0.3198	0.9404	0.038*	
O1	0.9299 (4)	0.2412 (3)	0.4017 (2)	0.0432 (6)	
N1	0.8176 (4)	0.1439 (3)	0.6778 (2)	0.0310 (6)	
O2	0.7771 (4)	0.5056 (3)	0.4308 (2)	0.0463 (6)	
O4	0.4586 (4)	0.6330 (3)	0.8526 (2)	0.0511 (7)	
C3	0.6630 (5)	0.4031 (4)	0.7370 (3)	0.0309 (7)	
C5	0.7482 (5)	0.1401 (4)	0.8172 (3)	0.0295 (7)	
O5	0.7336 (3)	−0.0027 (3)	0.4303 (2)	0.0456 (6)	
H5A	0.6363	0.0901	0.4178	0.055*	
H5B	0.7239	−0.0620	0.3698	0.055*	
C4	0.5565 (5)	0.5820 (4)	0.7409 (3)	0.0359 (8)	
N3	0.7778 (4)	−0.1290 (3)	1.1669 (3)	0.0395 (7)	
C1	0.8292 (5)	0.3538 (4)	0.4769 (3)	0.0358 (8)	
C10	0.7591 (5)	−0.0006 (4)	1.0681 (3)	0.0357 (7)	
H10	0.7395	0.0988	1.1006	0.043*	
C2	0.7669 (5)	0.3071 (4)	0.6286 (3)	0.0307 (7)	
C6	0.7672 (5)	−0.0064 (4)	0.9188 (3)	0.0302 (7)	
C9	0.8085 (5)	−0.2721 (4)	1.1191 (3)	0.0408 (8)	
H9	0.8238	−0.3634	1.1865	0.049*	
C8	0.8181 (5)	−0.2887 (4)	0.9732 (3)	0.0413 (8)	
H8	0.8411	−0.3901	0.9436	0.050*	
C7	0.7934 (5)	−0.1542 (4)	0.8716 (3)	0.0381 (8)	
H7	0.7943	−0.1628	0.7732	0.046*	

### Atomic displacement parameters (Å^2^)

**Table:**

	*U*^11^	*U*^22^	*U*^33^	*U*^12^	*U*^13^	*U*^23^
Co1	0.0425 (4)	0.0304 (4)	0.0199 (3)	0.0005 (3)	0.0020 (2)	−0.0042 (2)
O3	0.0634 (17)	0.0283 (12)	0.0362 (13)	−0.0031 (12)	0.0034 (12)	−0.0062 (10)
N2	0.0357 (15)	0.0312 (14)	0.0193 (12)	−0.0042 (12)	0.0043 (10)	−0.0056 (10)
O1	0.0519 (15)	0.0365 (13)	0.0219 (11)	0.0020 (11)	0.0086 (10)	−0.0030 (10)
N1	0.0381 (15)	0.0277 (13)	0.0184 (12)	−0.0038 (12)	0.0022 (10)	−0.0016 (10)
O2	0.0629 (16)	0.0325 (13)	0.0277 (12)	−0.0056 (12)	0.0065 (11)	0.0050 (10)
O4	0.0706 (18)	0.0352 (13)	0.0320 (13)	0.0002 (12)	0.0022 (12)	−0.0142 (10)
C3	0.0337 (17)	0.0270 (16)	0.0239 (15)	−0.0026 (13)	0.0012 (12)	−0.0043 (12)
C5	0.0319 (17)	0.0280 (16)	0.0217 (14)	−0.0035 (13)	0.0014 (12)	−0.0047 (12)
O5	0.0465 (14)	0.0443 (14)	0.0339 (12)	−0.0005 (11)	−0.0039 (10)	−0.0109 (10)
C4	0.0436 (19)	0.0300 (17)	0.0261 (16)	−0.0045 (15)	−0.0011 (14)	−0.0043 (14)
N3	0.0473 (17)	0.0373 (16)	0.0261 (13)	−0.0089 (13)	0.0000 (12)	0.0001 (12)
C1	0.0409 (19)	0.0346 (18)	0.0198 (15)	−0.0019 (15)	0.0016 (13)	−0.0014 (13)
C10	0.0412 (19)	0.0343 (18)	0.0261 (15)	−0.0083 (15)	0.0008 (13)	−0.0044 (13)
C2	0.0357 (18)	0.0269 (16)	0.0217 (14)	−0.0036 (14)	0.0010 (12)	−0.0026 (12)
C6	0.0311 (17)	0.0298 (16)	0.0227 (14)	−0.0055 (13)	0.0047 (12)	−0.0025 (12)
C9	0.043 (2)	0.0353 (18)	0.0373 (18)	−0.0102 (16)	−0.0024 (15)	0.0064 (15)
C8	0.050 (2)	0.0316 (18)	0.0390 (19)	−0.0113 (16)	0.0023 (15)	−0.0070 (15)
C7	0.044 (2)	0.0421 (19)	0.0241 (15)	−0.0118 (16)	0.0048 (14)	−0.0074 (14)

### Geometric parameters (Å, °)

**Table:**

Co1—O5^i^	2.061 (2)	C3—C2	1.374 (4)
Co1—O5	2.061 (2)	C3—C4	1.481 (4)
Co1—O1	2.098 (2)	C5—C6	1.465 (4)
Co1—O1^i^	2.098 (2)	O5—H5A	0.8500
Co1—N1	2.215 (2)	O5—H5B	0.8499
Co1—N1^i^	2.215 (2)	N3—C10	1.332 (4)
O3—C4	1.279 (4)	N3—C9	1.339 (4)
O3—H3	0.8200	C1—C2	1.479 (4)
N2—C5	1.358 (4)	C10—C6	1.394 (4)
N2—C3	1.368 (4)	C10—H10	0.9300
N2—H2	0.8600	C6—C7	1.384 (4)
O1—C1	1.243 (4)	C9—C8	1.381 (4)
N1—C5	1.334 (3)	C9—H9	0.9300
N1—C2	1.374 (4)	C8—C7	1.380 (4)
O2—C1	1.276 (4)	C8—H8	0.9300
O4—C4	1.223 (4)	C7—H7	0.9300
			
O5^i^—Co1—O5	180.0	Co1—O5—H5A	115.6
O5^i^—Co1—O1	89.88 (10)	Co1—O5—H5B	127.2
O5—Co1—O1	90.12 (10)	H5A—O5—H5B	107.7
O5^i^—Co1—O1^i^	90.12 (10)	O4—C4—O3	124.5 (3)
O5—Co1—O1^i^	89.88 (10)	O4—C4—C3	119.0 (3)
O1—Co1—O1^i^	180.0	O3—C4—C3	116.5 (3)
O5^i^—Co1—N1	90.05 (9)	C10—N3—C9	117.7 (3)
O5—Co1—N1	89.95 (9)	O1—C1—O2	124.0 (3)
O1—Co1—N1	78.08 (8)	O1—C1—C2	117.3 (3)
O1^i^—Co1—N1	101.92 (8)	O2—C1—C2	118.7 (3)
O5^i^—Co1—N1^i^	89.95 (9)	N3—C10—C6	123.8 (3)
O5—Co1—N1^i^	90.05 (9)	N3—C10—H10	118.1
O1—Co1—N1^i^	101.92 (8)	C6—C10—H10	118.1
O1^i^—Co1—N1^i^	78.08 (8)	N1—C2—C3	110.8 (2)
N1—Co1—N1^i^	180.00 (7)	N1—C2—C1	119.0 (3)
C4—O3—H3	109.5	C3—C2—C1	130.2 (3)
C5—N2—C3	108.6 (2)	C7—C6—C10	117.7 (3)
C5—N2—H2	125.7	C7—C6—C5	121.9 (3)
C3—N2—H2	125.7	C10—C6—C5	120.4 (3)
C1—O1—Co1	117.58 (19)	N3—C9—C8	122.3 (3)
C5—N1—C2	105.3 (2)	N3—C9—H9	118.9
C5—N1—Co1	146.4 (2)	C8—C9—H9	118.9
C2—N1—Co1	107.94 (17)	C7—C8—C9	119.7 (3)
N2—C3—C2	104.8 (3)	C7—C8—H8	120.1
N2—C3—C4	121.4 (3)	C9—C8—H8	120.1
C2—C3—C4	133.6 (3)	C8—C7—C6	118.7 (3)
N1—C5—N2	110.5 (2)	C8—C7—H7	120.6
N1—C5—C6	126.5 (3)	C6—C7—H7	120.6
N2—C5—C6	123.0 (2)		
			
O5^i^—Co1—O1—C1	−88.7 (3)	Co1—O1—C1—C2	−0.4 (4)
O5—Co1—O1—C1	91.3 (3)	C9—N3—C10—C6	0.8 (5)
N1—Co1—O1—C1	1.4 (2)	C5—N1—C2—C3	−1.2 (4)
N1^i^—Co1—O1—C1	−178.6 (2)	Co1—N1—C2—C3	−176.6 (2)
O5^i^—Co1—N1—C5	−84.2 (4)	C5—N1—C2—C1	178.0 (3)
O5—Co1—N1—C5	95.8 (4)	Co1—N1—C2—C1	2.6 (3)
O1—Co1—N1—C5	−174.0 (4)	N2—C3—C2—N1	1.3 (4)
O1^i^—Co1—N1—C5	6.0 (4)	C4—C3—C2—N1	−172.9 (3)
O5^i^—Co1—N1—C2	87.8 (2)	N2—C3—C2—C1	−177.8 (3)
O5—Co1—N1—C2	−92.2 (2)	C4—C3—C2—C1	8.0 (6)
O1—Co1—N1—C2	−2.0 (2)	O1—C1—C2—N1	−1.7 (5)
O1^i^—Co1—N1—C2	178.0 (2)	O2—C1—C2—N1	178.3 (3)
C5—N2—C3—C2	−0.9 (3)	O1—C1—C2—C3	177.3 (3)
C5—N2—C3—C4	174.2 (3)	O2—C1—C2—C3	−2.7 (6)
C2—N1—C5—N2	0.6 (4)	N3—C10—C6—C7	0.9 (5)
Co1—N1—C5—N2	172.7 (3)	N3—C10—C6—C5	−179.1 (3)
C2—N1—C5—C6	−179.1 (3)	N1—C5—C6—C7	−23.7 (5)
Co1—N1—C5—C6	−7.0 (6)	N2—C5—C6—C7	156.7 (3)
C3—N2—C5—N1	0.2 (4)	N1—C5—C6—C10	156.4 (3)
C3—N2—C5—C6	179.9 (3)	N2—C5—C6—C10	−23.3 (5)
N2—C3—C4—O4	−0.4 (5)	C10—N3—C9—C8	−0.9 (5)
C2—C3—C4—O4	173.0 (4)	N3—C9—C8—C7	−0.7 (5)
N2—C3—C4—O3	−179.9 (3)	C9—C8—C7—C6	2.4 (5)
C2—C3—C4—O3	−6.5 (6)	C10—C6—C7—C8	−2.5 (5)
Co1—O1—C1—O2	179.7 (3)	C5—C6—C7—C8	177.6 (3)

Symmetry codes: (i) −*x*+2, −*y*, −*z*+1.

### Hydrogen-bond geometry (Å, °)

**Table:**

*D*—H···*A*	*D*—H	H···*A*	*D*···*A*	*D*—H···*A*
O3—H3···O2	0.82	1.65	2.465 (3)	176.
N2—H2···O4^ii^	0.86	2.00	2.840 (3)	166.
O5—H5A···O3^iii^	0.85	2.07	2.918 (3)	173.
O5—H5B···N3^iv^	0.85	2.02	2.784 (3)	150.

Symmetry codes: (ii) −*x*+1, −*y*+1, −*z*+2; (iii) −*x*+1, −*y*+1, −*z*+1; (iv) *x*, *y*, *z*−1.
